# Reward-Induced Phasic Dopamine Release in the Monkey Ventral Striatum and Putamen

**DOI:** 10.1371/journal.pone.0130443

**Published:** 2015-06-25

**Authors:** Kenji Yoshimi, Shiori Kumada, Adam Weitemier, Takayuki Jo, Masato Inoue

**Affiliations:** 1 Department of Neurophysiology, Juntendo University School of Medicine, Bunkyo-ku, Tokyo, Japan; 2 Department of Psychology, Japan Women's University, Kawasaki, Kanagawa, Japan; 3 RIKEN BSI, Wako, Saitama, Japan; 4 Department of Neurology, Juntendo University School of Medicine, Bunkyo-ku, Tokyo, Japan; Chiba University Center for Forensic Mental Health, JAPAN

## Abstract

In-vivo voltammetry has successfully been used to detect dopamine release in rodent brains, but its application to monkeys has been limited. We have previously detected dopamine release in the caudate of behaving Japanese monkeys using diamond microelectrodes (Yoshimi 2011); however it is not known whether the release pattern is the same in various areas of the forebrain. Recent studies have suggested variations in the dopaminergic projections to forebrain areas. In the present study, we attempted simultaneous recording at two locations in the striatum, using fast-scan cyclic voltammetry (FSCV) on carbon fibers, which has been widely used in rodents. Responses to unpredicted food and liquid rewards were detected repeatedly. The response to the liquid reward after conditioned stimuli was enhanced after switching the prediction cue. These characteristics were generally similar between the ventral striatum and the putamen. Overall, the technical application of FSCV recording in multiple locations was successful in behaving primates, and further voltammetric recordings in multiple locations will expand our knowledge of dopamine reward responses.

## Introduction

Voltammetric detection of behaviorally-correlated dopamine release in the non-human primate brain would serve as a valuable experimental model of drug addiction and Parkinson’s disease. However, to date, successful systematic detection of cue- and reward-induced phasic dopamine release in behaving primates has been limited **[1, 2 and 3].** In our study **[1]**, we were able to detect reward-induced phasic dopamine release in the caudate striatal region using amperometry with boron-doped diamond electrodes as probes. In the current study we report our efforts to record dopaminergic reward responses in multiple locations in the primate striatum using fast-scan cyclic voltammetry (FSCV) with carbon fiber microelectrodes.

Voltammetric studies in rodents have revealed regional variation in reward-related dopamine responses **[4, 5, 6, 7 and 8]**. These differences may be derived from the topographical organization of projections from differentially-responsive dopamine neurons**,** or local variations in release regulation by differential transporter distribution **[9, 10]**. Apparent differences in response kinetics between ventral striatum and putamen suggest selective sensitivity to particular components of reward stimuli, such as reward value and reward timing **[11, 12 and 13].** Also, local differences in striatal cholinergic tonically active neurons (TAN) can cause variance in dopamine neurotransmission **[14, 15 and 16]**.

Studies in midbrain dopaminergic unit firing in primates have suggested variable characteristics of dopamine release throughout the primate striatum **[17]**. However, unlike rodents, the projection distribution of primate midbrain dopamine neurons is not as clearly segregated within projection areas **[9, 10]**. Therefore, without local recordings of phasic dopamine release, it is difficult to identify which parts of the primate brain receive these differential dopamine signals that are recorded in the midbrain.

In our previous study **[1]** we developed a physically durable diamond microelectrode for voltammetric recording in large brains. However, there were two limitations for the use of diamond microelectrodes. First, recording with constant-potential amperometry is vulnerable to noise, and second, the sensitivity was often lost in the brain tissue. For simultaneous multiple recordings, we decided to use carbon fibers because diamond microelectrodes had a high risk of sensitivity loss for unknown reasons. The high sensitivity of carbon fiber electrodes to dopamine, and their capacity for electrical recordings of impulse activity **[18]** make them useful for combined studies with conventional electrophysiology and electrochemistry.

In this article, we share our recent technical challenges and successes in monkey voltammetry. Because our initial attempts failed to detect relevant phasic changes in our first animal, several modifications in electrodes, behavioral procedures, and voltammetric techniques were tried to find the best experimental condition to detect reliable reward responses. While our report represents primarily technical advancement, it now permits the detection of statistically significant event-related phasic dopamine changes in more than half of the recordings. We believe that this technique is now sufficiently advanced to undertake an analysis of actual dopamine release by behavioral events at multiple locations in the monkey brain.

## Materials and Methods

### 2–1. Animals and care

#### 2-1-1. Animals

Three Japanese monkeys (monkeys U, C and S; Macaca fuscata, female, 5 to 7 kg) participated in the voltammetry experiments. Monkey U and S were used for MFB stimulation, and S and C were for behavioral tasks (experiments S1-8 and C1-7 in Tables 1 and 2). The brain from one additional male monkey, which was euthanized after our similar experiment, was used for immunohistochemical staining of dopamine neurons. One monkey was provided by Kawahara Bird-animal Trading (Tokyo, Japan), and two were provided by NBRP "Japanese Monkeys" through the National BioResource Project (NBR) of the MEXT, Japan. K.Y. and M.I. are approved for certificate of NBR primate care instruction course (#380 and #358 respectively). The procedures were conducted in accordance with the Guidelines for Proper Conduct of Animal Experiments established by the Science Council of Japan, and all experiments were approved by the Ethics Review Committee for Animal Experimentation of Juntendo University School of Medicine (protocol number 935). All possible efforts were made to minimize the number of animals used and their suffering. Throughout the study, animals were monitored in consultation with the institution's clinical veterinarian and also medical doctors of Department of Neurology of Juntendo University School of Medicine. No animals were euthanized during this study.

**Table 1:** Lick response ratio (%).

Exp	free	CS+	CS-	
	(no CS)	(red)	(blue)	
S2	100.0	93.3	26.7	**
S3	90.0	72.7	58.3	
S5	61.1	38.9	50.0	
S6	42.1	70.6	42.1	
S7	77.8	21.7	65.2	##
S8	100.0	100.0	58.6	**
average	78.5	66.2	50.2	
C1	63.0	74.1	17.9	**
C2	100.0	100.0	21.1	**
C4	100.0	97.3	21.1	**
C5	89.5	92.1	27.8	**
C6	88.9	89.2	57.9	*
C7	100.0	97.4	38.9	**
average	90.2	91.7	30.8	

Rate of licking responses from the presentation of timing cue (white dot, 2.5s before the juice delivery/CS-offset to 2.5s after) is summarized (See also Fig 3C). For comparison, the response of free trials during the same time window (from -2.5 to +2.5s from the juice onset) is also shown. While the response rate after the CS+ (red circle) was consistently higher than CS- (blue square) in monkey C, the difference was not clear in monkey S, except S2 and S8. High response rate of free trials indicate the animals could detect the juice delivery without preceding signals.

* p<0.05,

**: p < 0.01,

##: p<0.01; Difference of response ratio between CS- and CS+ (Chi-square test). Note opposite preference in exp S7. Exp S8 was done 6 months after S7.

**Table 2:** Summary of event-related temporal changes of dopamine-like component.

		Before Prediction time	juice cue t x CS	After Juice time	juice after CS t x CS	Free Juice	Handed Food	
	S5	***	*	-	-	-	***	
	S6	-	-	***	-	-	***	
	S7	-	-	-	**	-	###	
Ventral	S8	-	-	-	*	*	***	
Striatum	C1	-	-	-	-	**	-	
	C2	***	-	-	**	***		
	C4	***	-	*	-	***	*	
	C5	-	-	###	-	-	-	
	C6	***	*	-	***	***		
	C7	***	-	-	-	**		
Rate of positive response		5/10	2/10	2/10	4/10	6/10	4/7	
	S3	-	-	-	*	-		
	S5	*	-	-	-	*		X
	S6	-	-	-	*	-	*	
Putamen	S7	-	-	-	*	-	###	
	C2	***	-	-	-	**		
	C4	***	-	*	*	***	**	
	C5	**	-	-	-	***	##	
	C6	***	-	-	-	***		
	C7	***	-	-	***	***		
Rate of positive response		6/9	0/9	1/9	5/9	6/9	2/4	

*: p<0.05,

**: p<0.01,

***: p<0.001 for positive responses.

#: p<0.05,

##: p<0.01,

###: p<0.001 for negative responses.

Summary of ANOVA results. Prediction cue responses (before juice delivery) were calculated for the time window of -2.9 to 0.0 s from juice onset, where the timing cue appeared from -2.5 to -1.5 s and discriminative CS appeared from -1.5 to 0.0 s. Juice responses were calculated for the time window of -0.4 to 2.5 s from juice onset. For food delivery analysis, -2.9 to 3.0 s from contact to the mouth. Blanks in the handed food column indicate not tested. The food response in the S5 putamen was discarded (x) because the unstable line connection of the putamen electrode at the end of the experiment. The effect of time before juice delivery indicates a temporal change in the response to the prediction cue, and the time x CS interaction (t x CS) indicates a differential response to CS+ (red) and CS- (blue). The effect of juice over time indicates a temporal change in the response to juice delivery, and the time x CS interaction (t x CS) indicates a differential response to the presence or absence of juice delivery.

#### 2-1-2. Daily care

Animals were housed individually in 70x70x80cm cages and were on 12 hour light/dark cycle. Animals followed a primate diet (PS-A Oriental Yeast CO.LTD) and received daily sliced sweet potato. Animals also had access to primate toys and biting wood (S1 Fig), which were rotated on a weekly basis. Our monkey facility had wide windows, and natural sunlight lit the room beside 12 hr light-dark illumination 0700 to 1900. Animals had partial access to the outside view from the 11th floor window. Animal rooms are set to a constant temperature of 24 deg Celsius and the moisture was set 40 to 60%.

On weekday mornings, general behavioral responses of the animals were observed while offering small pieces of soft biscuit. At least three times a week, the animal was moved to the monkey chair to inspect and clean the head chamber with saline and treated with antibiotic ointment (gentacin or ofloxacin). The ointment was also spread on the border of the skin and dental cement, and the body weight was recorded. Water restriction was minimized for just two days immediately before behavioral voltammetric recordings (150ml/day). On the other days, a 500ml water bottle was given at meal-time or after the training. Water and monkey food chow (PS-A Oriental Yeast CO., LTD. Tokyo Japan) was given around noon, unless no other treatments or trainings were made. Thus, animals are monitored at least twice daily by lab personnel on weekdays.

#### 2-1-3. Anesthetic and analgesic procedures

**Fig 1:**
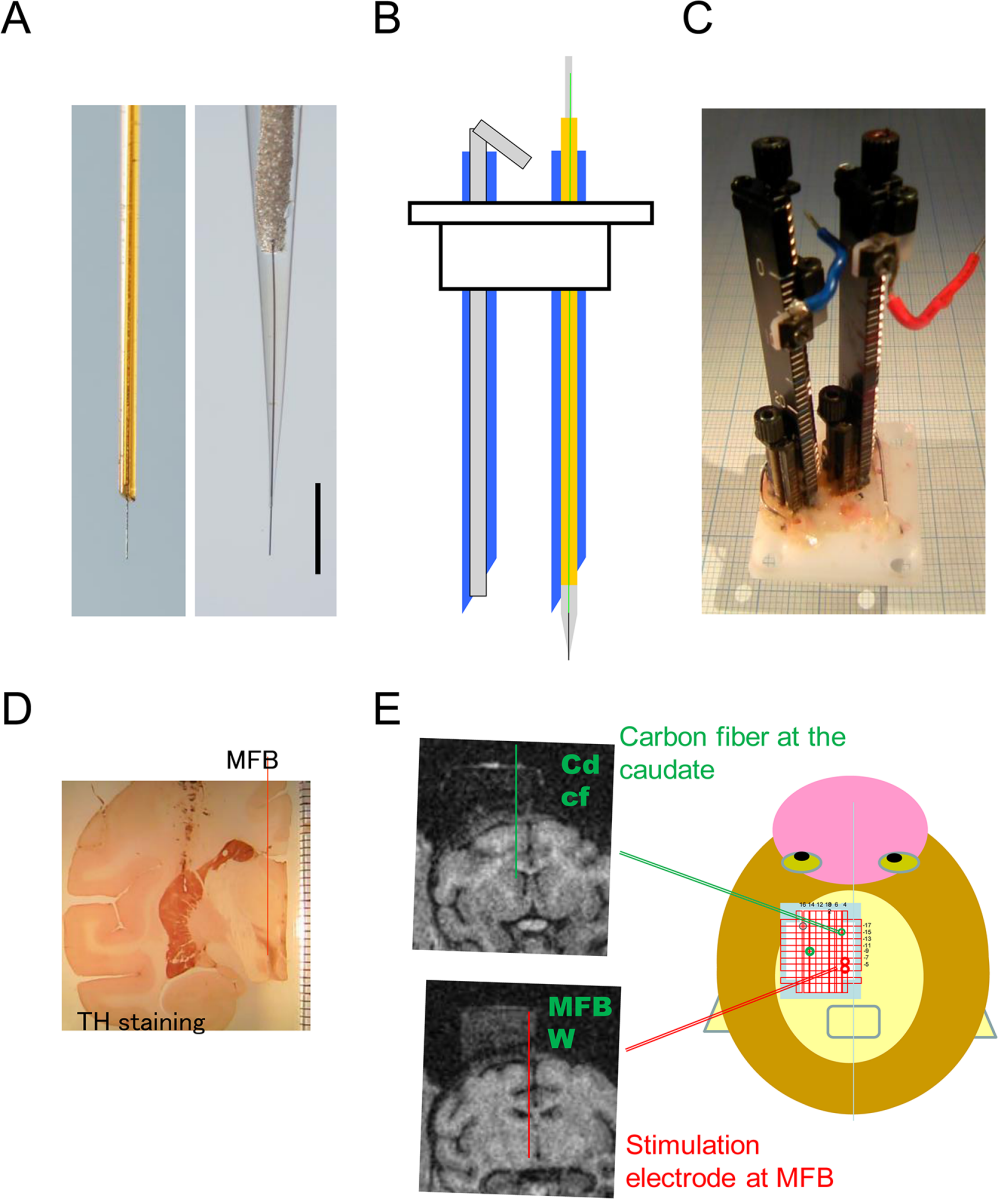
Electrode positioning. (A) Carbon fiber microelectrodes. Left: First design of microelectrode coated by a silica tube with 0.09 mm diameter, used in part of the early MFB stimulation experiments. Right: Microelectrode coated by a glass capillary, used for the most recordings in this study. Scale bar = 0.5 mm. Note the diameter next to the carbon fiber is thinner in the glass design (right). (B) Schematic drawing of the guide cannula (blue) (OD 0.7 mm, ID 0.42 mm). Left: An inset was placed to protect the inside of the tube when inserting into the brain. Right: The inset was replaced by the elongated carbon fiber microelectrode with a shaft of silica tube of 0.35 mm diameter. (C) Grid with vertical holes of 0.7 mm diameter and two manual-drive micromanipulators. (D) Coronal section immunostained with tyrosine hydroxylase (TH) showing the position of the striatum and MFB. (E) Schematic drawing of the grid on the monkey’s head. The electrode path is shown on the left MRI image. Note the electrode chamber on the skull, as visualized by filling with 2% agar.

All surgical procedures were performed under general anesthesia using aseptic techniques. A chronic recording chamber and a head holder were implanted in the monkey skull (Fig 1E). The chamber was placed in either the right (monkey U and S) or left (monkey C) striatum. Before surgery, the animals were pretreated with 0.015mg/kg of atropine and anesthesia was induced with ketamine-HCl (4 mg/kg, i.m.) and xylazine (1 mg/kg, i.m.). Antibiotics (cefazolin 50mg/body, i.m.) and diclofenac sodium suppository were given. Heart rate and SPO2 monitors were attached immediately before placement of the animal in the stereotaxic frame. An intravenous catheterization was made and pentobarbital sodium (10 mg/kg, i.v.) and butorphanol (0.03mg/kg, i.v.) were injected slowly to maintain anesthesia during the surgery. A recording chamber (25 x 30 mm) and a head-post holder were attached with dental cement and titanium anchor screws. Craniotomy was made under the recording chamber. Following surgical procedures, animals were monitored and given antibiotics (cefazolin 50 mg/body, i.m.) for 5 days. No scleral search coil to monitor eye position was implanted in this study.

Coronal MRI images of the head and brain were taken prior to the experiment with a 0.3-T magnetic resonance scanner (AIRIS2, Hitachi Medical Co., Tokyo, Japan) to determine the position of implantation for each monkey. For MRI scanning, animals were anesthetized with ketamine-HCl (4mg/kg, i.m.) and medetomidine (0.15mg/kg, i.m.). The chamber was visualized on MRI image by filling either with 2% agar or ointment with paraffin liquid. The relative position of the chamber was determined from the MRI images. Unit recordings were also used to verify the depth from the chamber.

During electrical stimulation of MFB, anesthesia was induced with ketamine-HCl (2 mg/kg, i.m.) and medetomidine (0.02 mg/kg, i.m.) and maintained under general inhalational anesthesia with 0.5–2% isoflurane in 30% O2 and 70% N2O. Heart rate and SPO2 was monitored until starting the recordings. N2O gas was turned off more than 10 min before releasing the animal.

### 2–2. Electrodes (Fig 1)

Carbon fiber was supported by two ways, silica tube or glass capillary (Fig 1A). Silica tube design (0.09 mm outer diameter) was used only in very early recordings of MFB stimulations and replaced by a glass capillary design, because accumulation of tissue debris blocked a part of carbon fiber surface at the blunt end of the silica tube. Carbon fiber microelectrodes of glass capillary design were prepared as previously described **[19, 20]**, except the shaft was elongated with a silica tube. These elongated electrodes are now commercially available (Huso Electrochemical Systems, Kawasaki, Japan). Individual carbon fibers (7 μm in diameter, HTA-7, Toyo-RENAX, Tokyo, Japan) were connected to a urethane-coated thin wire (0.08 mm diameter) with silver glue (DOTITE D-500, Fujikura Kasei, Tochigi, Japan), and the connection was sealed in pulled glass capillary tubes of 0.35 mm diameter with epoxy-resin (Cemedine 1565, Cemedine Co., Ltd, Tokyo, Japan), such that 300 μm of the carbon fibers protruded from the capillary tubes. The relatively long carbon fiber protrusion allowed for improved sensitivity. The glass capillary was elongated with a silica tube (0.35 mm outer diameter, GL Science, Tokyo, Japan). As the insulation of the electrode rod was essential, a thin wire insulated with urethane was used to connect the carbon fiber to the potentiostat though the silica tube. The end of the thin wire was soldered to a short thicker wire to make a reliable connection with the potentiostat. The potentiostat was clamped to a micromanipulator (MO-903, Narishige, Tokyo Japan) (Fig 1C), which also functioned as the terminal of the potentiostat. The tight connection of the thin wire was important to avoid the noise associated with vigorous behavior.

### 2–3. Positioning of the electrodes

**Fig 2:**
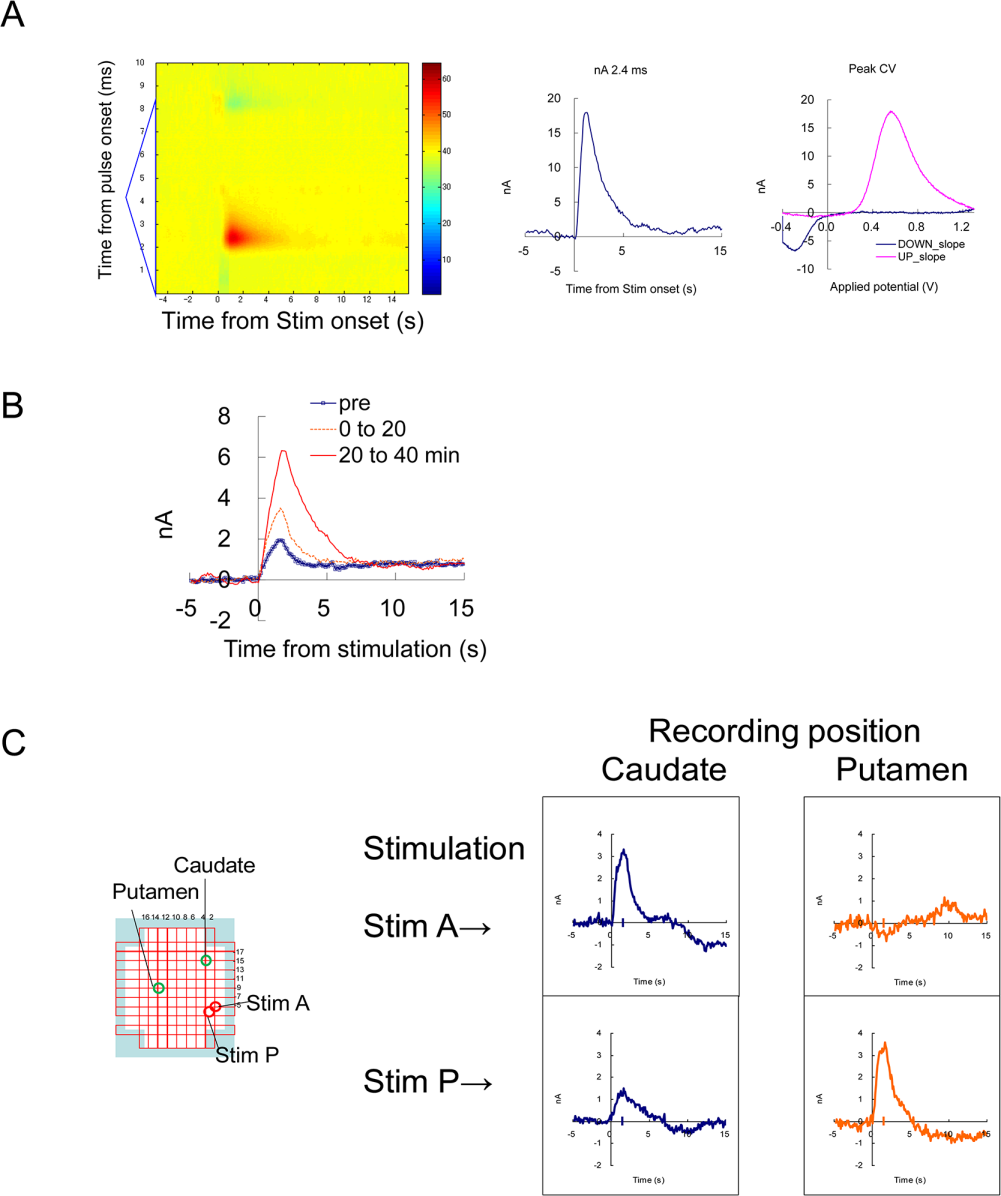
Evoked dopamine release by electrical stimulation. (A) An example of FSCV recording with MFB stimulation after nomifensine administration. Electrical stimulation of 60Hz, 48 pulses, was given at 5 s from the beginning of the recording. Left: A background-subtracted color plot showing a whole voltage scan from -0.4 to 1.3 V triangular waveform. Middle, Temporal current change at the dopamine peak potential. Right: Cyclic voltammogram from -0.4 to 1.3 V and back to -0.4 V, at the peak of the response. (B) Enhancement by a dopamine uptake inhibitor. MFB stimulation (30 Hz 24pulses) was repeated every 3 min, and an average of 6–7 responses before (pre), up to 20 min (0–20) and 20–40 min after the administration of nomifensine (1 mg/kg s.c.) were indicated. (C) Simultaneous recording from the caudate and putamen using the grid system. Two carbon fibers were placed as in Fig 1E, and two stimulating electrodes were placed in two locations near the MFB. Note that stimulation positions A and P were optimal for inducing responses in the caudate and the putamen, respectively. This result shows simultaneous and independent recording from two locations in the striatum.

The position of the electrode tips was determined using a rectangular grid system (Fig 1E). The plastic grid (AP 30 mm, ML 25 mm and 13 mm thick, Narishige, Tokyo, Japan) had grid of holes with 0.7 mm diameters. Each tip’s AP and ML position was determined by this grid, and the depth was determined by the micromanipulator on the grid. This grid system allowed the use of multiple electrodes simultaneously. For example, two carbon-fiber microelectrodes and two stimulation needles were set in the experiment shown in Fig 2C.

The electrode was placed into the brain through a steel guide cannula (0.7 mm O.D., 0.43 mm I.D. and 40 or 50 mm long). The cannula with the protection inset core inside was placed on a particular hole on the grid, and the tip of the cannula needle placed 2–5 mm above the recording target. Then, the inset was replaced by the elongated carbon fiber microelectrode under a surgical microscope.

The elongated carbon fiber was clamped by the micro-manipulator and advanced until a 30 mm mark on the shaft met the top of the known length of the cannula, then the depth on the manipulator was checked. When the carbon fiber depth reached the bottom of the cannula, neuronal impulse activity was recorded using a typical amplifier (MDA-4, BAK, MD, USA). The position was further adjusted until impulse activity adjacent to the carbon fiber was identified. The cannula and electrode were supported with glue to minimize the physical vibration of the electrodes.

Two Ag/AgCl wires were placed above the dura mater and used as reference and counter electrodes. After the in vivo recordings, the used carbon fiber microelectrode tips were immediately rinsed, kept in saline, and calibrated with dopamine solutions.

Two locations were selected for the voltammetric recordings in each monkey. The positions of the ventral striatum and putamen were determined using MRI images. The anterior positions of coronal slices were adjusted to a brain atlas **[21]**. The recording positions were anterior to interaural center +25.0 to 26.5, right 6.0, and 17 to 19 mm below the dural surface in the ventral striatum of monkey S; +22.5 anterior, left 2.5 to 4.0, and 18 to 26 mm below the dural surface in the ventral striatum of monkey C; +22.0 anterior, right 12.0, and 18 to 20 mm below the dural surface in the putamen of monkey S; and +18.0 to 19.5, left 10.0, and 20 to 22 mm below the dural surface in the putamen of monkey C.

MFB stimulation was attempted in two monkeys. The monkey was anaesthetized with ketamine and medetomidine and maintained with 1% isoflurane on a monkey chair. A stainless steel needle or tungsten electrode (FHC, Bowdin, ME, USA) was used to penetrate vertically, aiming at the medial edge of subthalamic nucleus (STN). The electrode was placed at the position of the thick bundle of tyrosine-hydroxylase-positive axons observed histologically at the medio-dorsal edge of the STN (Fig 1D), and electrical stimulation was delivered. For electrical stimulation, the dorso-ventral placement of the stimulating electrode was adjusted to obtain the maximal dopamine response. Biphasic constant current pulses were delivered to the MFB (30–60 Hz; biphasic 100–200 μA constant current, 24 pulses, 2 ms each) through the stimulating electrodes with an isolated stimulator (SS-202J connected to SEN-7203, Nihon-Koden, Co., Tokyo, Japan). The carbon fiber microelectrode was placed in the striatum, and the electrochemical response to the stimulation was recorded.

### 2–4. Electrochemical procedures

FSCV with a triangular waveform from -0.4 to 1.3V was mainly applied for electrochemical recordings in this study. In a part of sessions, rectangular-pulse voltammetry (RPV) was performed as described previously **[22]**. For simultaneous voltammetric recording at multiple locations, a counter electrode grounded type three-channel potentiostat was used (HECS-9139, Huso Electrochemical Systems, Kawasaki, Japan). Because simple potentiostat circuits set the working electrode potential at the ground level, they are not suitable for simultaneous multiple voltammetric recordings. Data acquisition was performed by a commercial control/recording system (TH-1; ESA Biosciences Inc., MA, USA) with two multifunction boards (NI-PCI-6221, National Instruments, TX, USA) implemented on a Windows PC (Epson, Endeavor). The simultaneous 4-channel recording was made as channel 0: ventral striatum; channel 1: putamen; channel 2: timing signal of CS and juice onset and channel 3: IR sensor of lip movement. Typically, the gain of the amplifier was 500 nA/V for FSCV and 50 nA/V for RPV. The time constant of the low-pass filter was 0.2 ms for FSCV and 2.0 ms for rectangular pulses. Voltage pulses were applied at 10 Hz. For in vitro evaluation of the current responses of chemicals, carbon fiber microelectrodes were placed in a flow of PBS, and the flow was switched to the test solutions **[1, 22]**.

For FSCV, a triangle waveform lasting for 8.5 ms was applied. The potential was ramped from -0.4 V to 1.3 V vs. the Ag/AgCl reference and back from 1.3 V to -0.4 V at 400 V/s. This triangle-positive wave gives the oxidation peak of dopamine at 2.5 ms from the beginning of the positive slope (appearing at the 600 mV position). For RPV, the potential was stepped from 0.0 V to 0.2 V for 30 ms. Dopamine detection was represented by averaged current value of 5 to 25 ms from the pulse onset (t5-25). The electrode was held at 0.0 V between the pulses. When a new waveform started for each method, the waveforms were applied for at least 3 min at 10Hz (1800 cycles) before starting the next measurement.

### 2–5. Behavioral tasks

#### 2-5-1. Apparatus

The animals were seated in a primate chair and well-habituated to the task in advance, with their heads held by the head holder. A tube was placed in front of the mouth for juice delivery (0.6 ml/0.6 s), which was electrically connected to ground at the same potential with the counter electrode of the animal. A 23-inch computer screen was placed 90 cm from the animal to deliver visual conditioning stimuli (CS). During the recordings, their behavior was videotaped. Trials were excluded from further analysis when the animal did not stay calm (described in detail below). Trials were delivered at randomized intervals of 10–20 s. The monkeys received 50 juice deliveries per day for 2–3 days per week, and voltammetric recording was performed once or twice a week. Water was restricted to 150 ml/day for two days before the recording and available ad libitum on the other days.

#### 2-5-2. Pavlovian procedure

**Fig 3:**
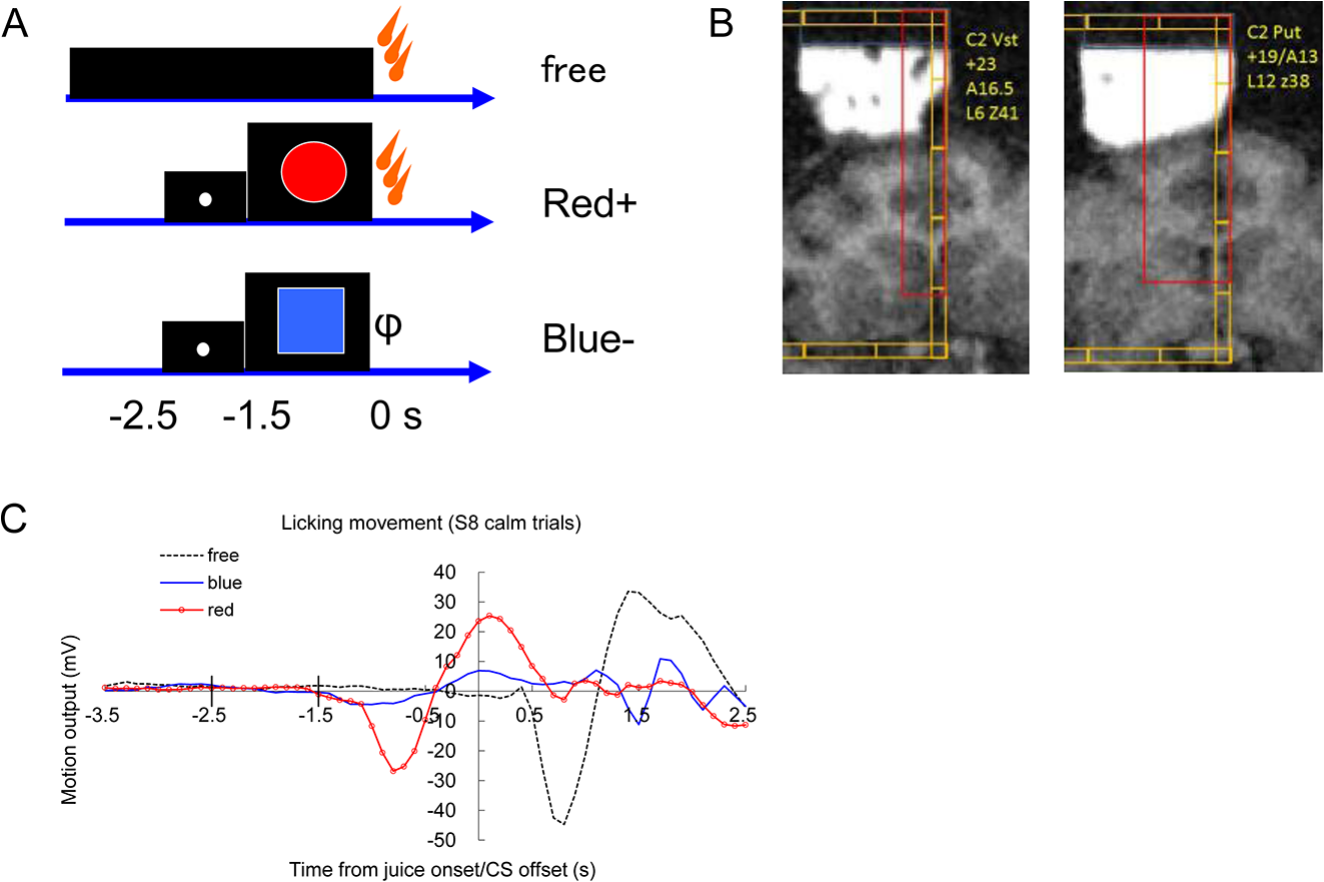
Liquid reward task and behavioral responses. (A) Pavlovian cue-reward task. A timing cue (white dot, -2.5 to -1.5 s) and CS+ (red circle, -1.5 to 0 s to liquid reward delivery) were presented on the screen in front of the monkeys. The blue square was not followed by a juice reward in ordinary sessions. Free juice was delivered without these visual cues. (B) Recording positions indicated on the MRI image. (C) Licking movement associated with the task. A representative example of experiment S8. Infra-red (IR) motion sensor output placed beside the mouth of the monkey. Only calm trials (97 out from 111 trials) without movements during the baseline (-5.4 to -2.5s) are averaged in this figure. Free (no CS): black dots, CS-: blue, CS+: red with circle. The abscissa indicates the time from juice onset/CS offset. Average of 25 to 49 trials in experiment S8.

The Pavlovian procedure was basically the same as that published by Matsumoto and Hikosaka in 2009 **[17]** (Fig 3A). At first, three conditioned stimuli (CS; red circle, green cross and blue square) were associated with a liquid reward (diluted orange juice) with 100%, 50% and 0% probability, respectively. The green cross with a 50% probability of reward was omitted for the last session of monkey S and all of monkey C. The liquid reward was delivered through a spout positioned in front of the monkey’s mouth. For all figures, the timings are presented with the juice onset as time 0. Each trial started after the presentation of a white dot as a timing cue (-2.5 s). After 1 s (-1.5 s), the timing cue disappeared, and one of the three conditioned stimuli was presented pseudo-randomly. The CS disappeared after 1.5 s (at 0 s), and the juice reward was delivered. In addition to the cued trials, un-cued trials were included in which a reward alone (free reward) was delivered. All trials were presented with a random inter-trial interval that averaged 15 s. One block consisted of 2 to 4 recording units of 5 min with fixed proportions of trial types (For trial blocks S1-6, free:blue:red:green-:green+ was 1:1:1:1:1, the probability of a juice reward 2.5 s after the white dot was 2/4; For S7 and C1-7, free:blue:red was 1:1:2, the probability of reward 2.5 s after white dot was 2/3). To monitor the monkeys’ licking behavior, we attached an infra-red sensor to the reward spout and measured the rapid IR change resulting from their licking **[14]**. The results of liquid reward trials were relatively consistent in our second animal (monkey C).

#### 2-5-3. Food trials

Small 0.5 g biscuits (Tamago-boro, Iwamoto Seika Co., Ltd., Aichi, Japan) were given to the animals restricted in the monkey-chair. The examiner (K.Y.) sat in front of the monkey face to face and took the pellets one by one from the backside table, approximately every 20 s. The delivery motion (look back, pick one biscuit, put it in front of the mouth of the monkey and activate a foot pedal timing-switch) occurred within 2 seconds. The body of the examiner was electrically connected to the common ground of the potentiostat. The food reward trials were effective to detect significant responses in our first animal (monkey S), instead of the typical Pavlovian liquid reward task.

### 2–6. Chemicals

Chemical reagents, including dopamine HCl (DA), serotonin HCl (5-HT), and adenosine were purchased from Sigma-Aldrich (St. Louis, MO, USA). Isoflurane was purchased from Abbott. The other special-grade reagents were purchased from WAKO (Tokyo, Japan).

### 2–7. Immunohistochemistry

A formaldehyde-fixed Japanese monkey brain was sliced into 40 μm coronal sections in a cryostat, and the sections were immunostained with an anti-TH rabbit antibody (Calbiochem) and visualized using the elite-ABC kit (Vector) and DAB as described previously **[23]**.

### 2–8. Statistical analysis

#### 2-8-1. Selective detection of dopamine signal

**Fig 4:**
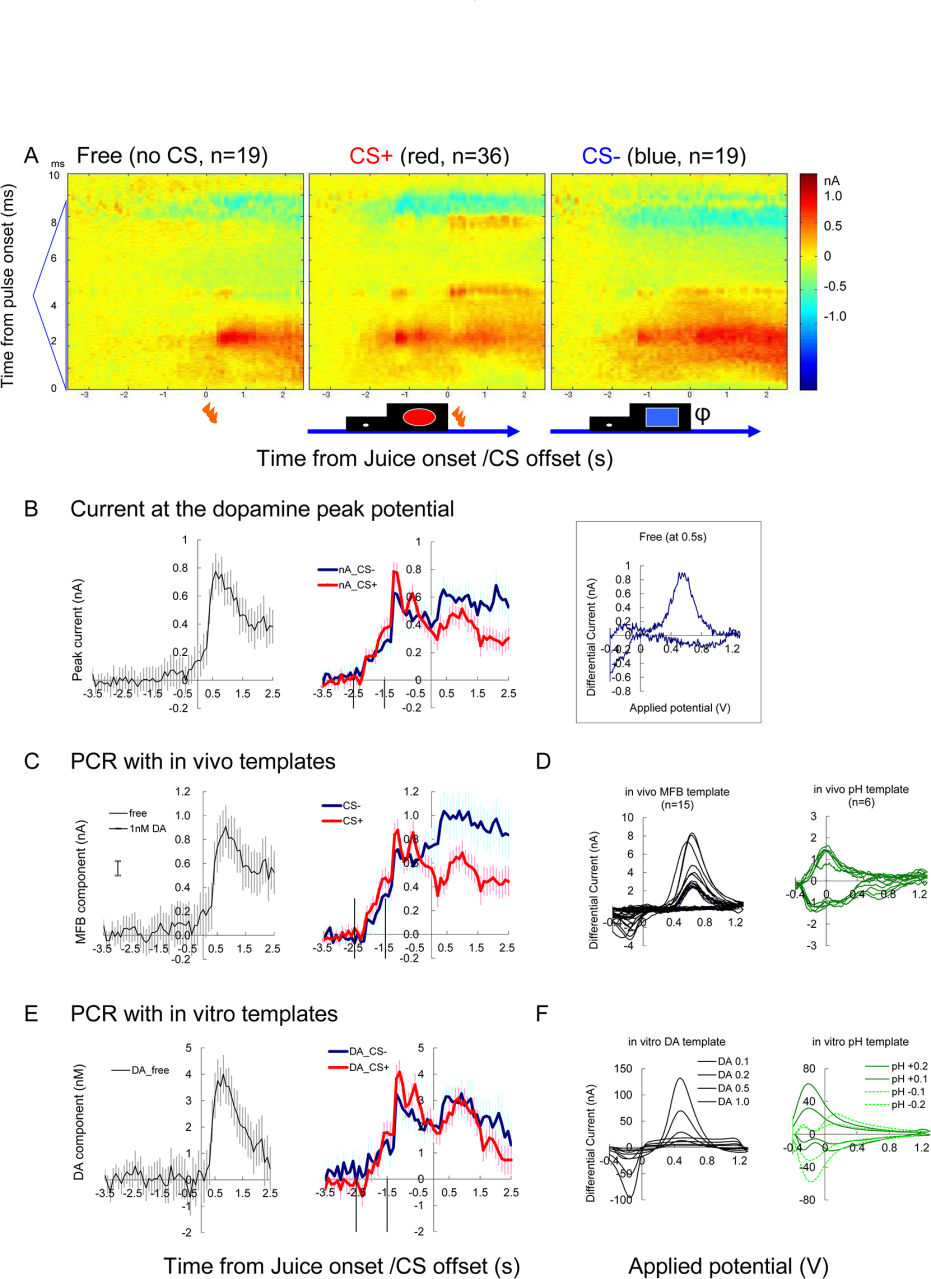
Representative example of juice reward task (Exp C4, putamen). (A) Color plot indicating the averaged differential voltammogram of free (left), CS+ (middle) and CS- (right) trials. Abscissa indicates time from juice delivery/CS offset. The ordinate indicates the time from the triangular pulse onset, from the bottom to the top. Note onset of positive response after the juice delivery in free trials (left). Similar response started at -1.5s in the CS+ and CS- trials (middle and right). (B) The current change at the dopamine oxidation peak potential (2.4 ms from the pulse onset). Free (black), CS+ (red with circle) and CS- (blue) trials. Average of 19 to 36 trials are shown with vertical bars indicating s.e.m. Inset on the right shows voltammogram 0.5 s after the free juice onset from averaged differential current (A left). (C) Dopamine-like component extracted by PCR analysis of the same data. (D) In-vivo template waveforms of dopamine and pH extracted from the MFB stimulation experiments as shown in Fig 2. (E) Same as C, but in-vitro template was used instead of in-vivo template. (F) Dopamine and pH waveforms obtained in-vitro, used for the analysis in E. Note the result value (ordinate) from in vivo template (D) is originally given as peak current (C, nA), while concentration (nM) value is directly given (E) from in vitro template (F). Vertical lines in B, C and E indicate onset of timing cue (-2.5s) and onset of CS (-1.5s)

The FSCV recording data were subjected to principal component regression analysis (PCR) **[24]**. The FSCV data were analyzed for each 10–20 min block of trials. First, using the timing file (channel 2), each trial was extracted as an 8-s time window (-5.5 to 2.5 s from the juice onset, time 0). The background waveform, averaging -5.5 to -2.5 s, was defined for each trial to prepare background-subtracted waveform data. Principal component regression was performed using an in-vivo dopamine-like and pH-like teaching template pooled from the MFB stimulation responses (Fig 4).

It is essential to exclude large current fluxes that can be induced by occasional frustrated struggling behavior of the animals. The extreme current “jump” caused by such behavior was prone to be induced when the electrodes and the manipulators were not fixed on the grid tightly. With the aid of physical tightening with epoxy-glue (High-speed Epo, Konishi, Osaka Japan) and also the innate robustness of FSCV recording, such current jump was rare compared to our previous study using constant-potential amperometry on diamond microelectrodes **[1]**. A cut off criteria of 5 nA was determined empirically to exclude all of the jump trials. The current at the dopamine oxidation peak (2.4 ms from the waveform onset) was extracted, and then the difference between the highest and lowest current during the 8-second window was calculated (max-min value) for each trial, and the trials with a max-min value exceeding 5nA were excluded (S2 Fig). The 5nA criteria seemed to be appropriate, although the criteria could be between 3 to 5 nA in our condition. A total of 10 trials were omitted from the further analysis in this study (S1 Table). Because the exclusion procedure was made on each electrode, the number of trials excluded may differ between the recording locations of the same block.

In most cases, the background of FSCV data is temporally sloped, which makes the analysis of temporal change difficult. This is derived from a continuous gradual maturation of the carbon fiber, which is most evident soon after starting to apply the waveform. More than 10 min of maturation period was taken before the first recordings of the day. After the background subtraction, exclusion of extreme data and PCR, the slope was adjusted so that the linear regression of the averaged pre-event background (-5.5 to -2.5 s) of the adjacent 40–80 trials in the session became temporally flat.

#### 2-8-2. Analysis of signal change over time

**Fig 5:**
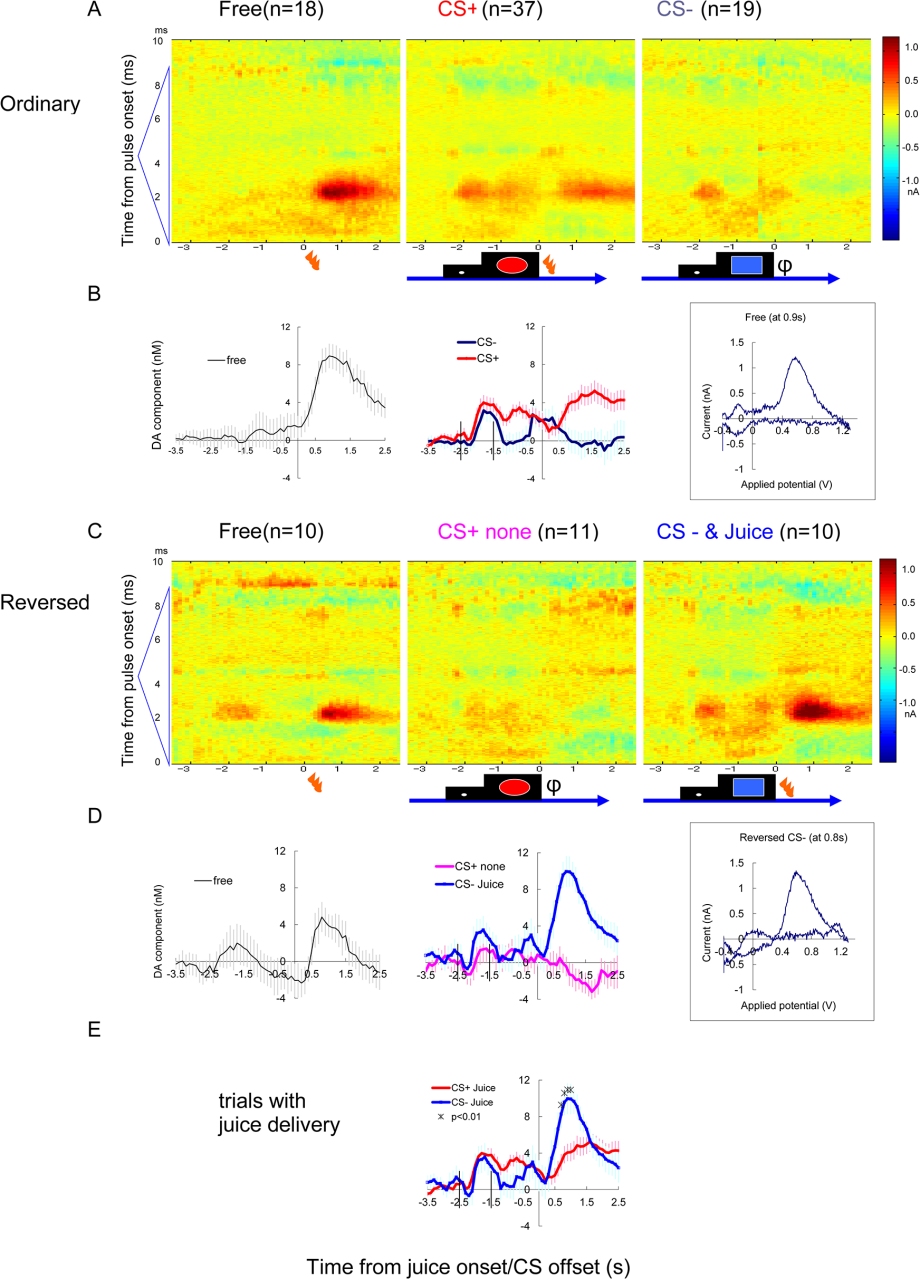
Reversed CS trial (Exp C6, ventral striatum). Additional example of FSCV recording from the ventral striatum. (A and B) Ordinary CS condition as shown in Fig 3. (B) Average of PCR result of 18 to 37 trials are shown with vertical bars indicating s.e.m. Inset on the right shows voltammogram 0.9 s after the free juice onset from averaged differential current (A left). (C and D) Reversed session in which juice was delivered following the ordinary CS- (blue square), instead of the ordinary CS+ (red circle). Average of 10 to 11 trials. Note that during the reversed session (C and D), a large response to juice delivery followed the ordinary CS-. Inset on the right in D shows voltammogram 0.8 s after the juice onset following CS- from averaged differential current (C right). A positive dopamine-like response was also induced by the timing cue (-2.5 to -1.5 s). (E) The direct comparison between actual juice delivery following ordinal CS+ and CS- (*: p <0.01, by t-test (two-tailed)). Vertical lines in B, D and E indicate onset of timing cue (-2.5s) and onset of CS (-1.5s)

**Fig 6:**
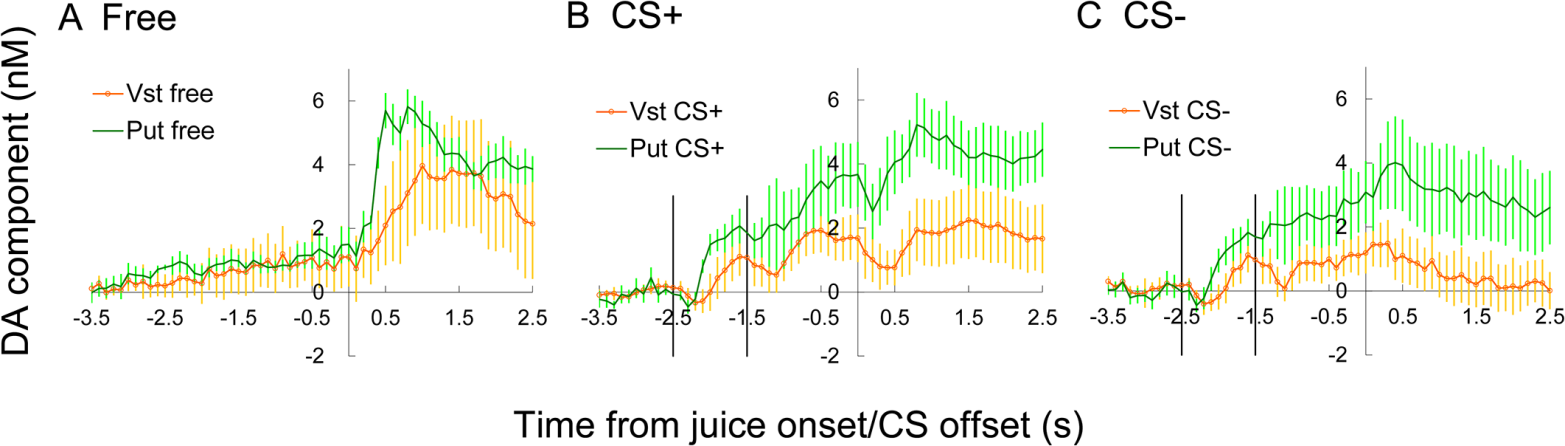
Summary of all paired recordings of monkey C. The average of all simultaneous recording pairs to compare the results from the ventral striatum and the putamen. The average of ventral striatum (orange with circle) and putamen (green solid line) are compared for free (A), CS+ (B) and CS- (C) trials. Five paired-recordings were subjected to ANOVA. No interaction with location was detected for the responses to a prediction cue or juice. Prediction cue (B and C, -2.9 to 0.0s): time F_(29,464)_ = 18.40, p < 0.001, interaction time with location was NS and the interaction of time and CS was NS. Juice after CS (B and C, -0.4 to +2.5s): The interaction between time and CS was significant F_(29,464)_ = 6.72 p = 0.002, but interaction of time with location was NS (F_(29,464)_ = 1.56). Free juice (A): the effect of time was significant F_(29,232)_ = 14.05 p < 0.001, the interaction of time with location was NS (F_(29,232)_ = 1.76)

For the ANOVA analysis of the temporal changes (Table 2), data from the trials was divided into separate time windows containing each event (CS or juice reward) to analyze the dopamine component over the course of a trial. The calculated dopamine-like component was aligned to the CS offset, which is also the onset of juice delivery (Figs 3, 4 and 5). The timing cue appeared at -2.5 to -1.5 s and the discriminative CS appeared at -1.5 to 0.0 s. Since voltammetric recording can only estimate the change from some time point, some benchmark level has to be appropriately defined. An average of 5 time points (0.5s) just before the event was subtracted as the benchmark. Analysis of the response to the CS included the -2.9 to 0.0 s time window, and the average of -2.9 to -2.5 s was subtracted as the benchmark. For the analysis of juice (US) responses, -0.4 to +2.5s was used and the average of -0.4 to 0.0s was regarded as the benchmark. For the food trials, the recording was aligned to the time of food contact to the mouth, and the time window of -2.9 to +3.0s was analyzed (Fig 6). The SPSS Ver17.0 software was used for ANOVA for repeated measure and the p value was determined after applying the Greenhouse-Geisser correction. The PCR value of dopamine-like component by in vivo teaching template was originally given as current (nA), and the dopamine concentration change (nM) was calculated using in vitro post-calibration result of the recovered carbon fiber electrode at 1000 nM dopamine (nM/nA).

## Results

### 3–1. Evoked dopamine release by electrical stimulation (Fig 2)

To validate the dopamine detection in the monkey brain, the evoked dopamine release in the striatum was recorded for monkeys U and S. First, to identify the location of the dopamine fiber bundle, frozen sections of Japanese monkey brain were immunostained with tyrosine hydroxylase (TH). The TH-positive medial forebrain bundle (MFB) was found at the medial border of the subthalamic nucleus (Fig 1D). Electrical stimulation of the MFB evoked dopamine-like responses in the striatum (Fig 2A). The response was enhanced by the administration of a dopamine uptake inhibitor (nomifensine, 1.0 mg/kg, s.c., Fig 2B). Evoked dopamine release was recorded from two monkeys. The optimal stimulation point for the caudate and putamen were determined (Fig 2C). A set of teaching template waveforms of in vivo dopamine release in the monkey striatum was prepared from these recordings. pH-like waveforms were also extracted 10–15 s after the stimulations.

### 3–2. Striatal dopamine release during reward tasks

The principal procedure was the CS-juice task with FSCV recording, which is summarized in this manuscript (Tables 1 and 2). The experiments were discarded if dopamine detection was not confirmed post-calibration of the carbon fiber recovered from the brain (S1, S4, C3), although this finding does not necessarily mean a lack of sensitivity during behavioral tasks. Noisy recordings due to poor connections of the wires were also discarded from the analysis (S2 and later part of S5 putamen).

We started with a typical Pavlovian task of liquid reward, following the previous studies **[11, 14, 17].** As is the nature of this type of trial and error for technical development, the task schedule was modified continuously and was thus partially inconsistent between experimental days, particularly for the first monkey, S. The licking responses, detected by an infra-red motion sensor beside the juice spout revealed clearly differentiated licking responses between CS+ and CS- trials in monkey C, but not in monkey S (Table 1). We confirmed differentiated behavior of Monkeys S at the beginning of this study, but the differentiated behavior was lost after experiment S2. The 50% probability CS trials (green cross), which was not shown in the daily training but introduced only in the recording sessions of monkey S, potentially confused monkey S so these trials were omitted from S7 and from all sessions with monkey C. The food trial was introduced from experiment S5. CS reversal was also tried in some of the experiments, but the technique was tried only four times to avoid the establishment of new reversed learning.

During the 15 recording days with behavioral tasks included in this article (February-October 2014), 33 elongated carbon fibers were used, 8 were snapped (4 above the guide-cannula), 4 were not sensitive to dopamine after recovery from the brain, and 3 were noisy for connection problems. One carbon fiber that half-snapped in the ventral striatum of C4 was included in the data for high remaining sensitivity to dopamine. In total, recordings by 19 electrodes in two female monkeys were analyzed, as shown below (Table 2).

### 3–3. Chemical identity of responses to free liquid reward

During the juice-reward sessions, juice delivery without a prediction cue (free juice) induced marked responses in many of the successful electrodes (12/19; Table 2). On the representative electrodes placed in the putamen (Fig 4) and ventral striatum (Fig 5), marked increase in dopamine-like signal is evident after the free juice delivery. The color plot of averaged differential waveforms of 10 to 36 trials of each trial type (Figs 4A and 5A) revealed positive current response similar to the response to MFB stimulation (Fig 2A). The current at the dopamine oxidation potential (2.4 ms from the onset of triangular waveform) shows a clear increase after the juice delivery (Fig 4A left).

In addition to the visual impression on the color plot, there are four good reasons supporting chemical identity of dopamine. First, the cyclic voltammograms at the juice response was roughly similar to dopamine (Figs 4B and 5B right inset). Second, PCR estimation of dopamine-component (Fig 4C), calculated using teaching templates prepared in vivo, was consistent to the peak current value at the dopamine peak potential (Fig 4B). Third, the result was consistent when the teaching template was replaced by pure dopamine and pH waveforms prepared in vitro (Fig 4E and 4F). These results indicate the validity and consistency of the voltammetric signals obtained in vivo during the tasks. Additionally, we used RPV in a few sessions (four sessions for S and three sessions for C). The successful example of RPV showed a similar pattern of results compared to FSCV (S4 Fig), although significant responses were observed less frequently than with FSCV, most likely due to a lower sensitivity.

Taking advantage of in vitro training sets, the data was further analyzed with in vitro templates of four pure chemicals, dopamine, pH, adenosine and serotonin (S3 Fig). The estimated change in serotonin-like component was temporally similar to dopamine, but the change was sustained longer. In the averaged color plot of CS+ responses (Fig 4A), positive current response at the peak of triangular applied potential (4.25 ms from the pulse onset) was observed. We have previously observed adenosine response at the peak of 1.3V triangular waveform **[22]**. Unique adenosine-like response was detected following CS+ (S3 Fig).

### 3–4. Responses to liquid reward in cue-reward tasks

The result of ANOVA analysis is summarized in Table 2. To compare the response to the actual juice reward delivery (US) with or without the prediction cue (CS), cue and reward responses were analyzed in separated time windows, as in the preceding electrophysiological studies **[11, 14, 17]**. The response to the juice delivery after the prediction cue was much less than the same juice delivery without a cue (Figs 4 and 5).

To verify the prediction dependency of the dopamine response clearly, reversed sessions were introduced in a few experiments (Fig 5). The difference between positive and negative juice delivery was modest when they followed after the ordinary CS (Fig 5B middle). However, when the CS was reversed transiently (Fig 5C and 5D), the response to the juice delivery after ordinary CS- (blue square) became equivalent to the free reward. Direct comparison of responses (Fig 5E) to the juice delivery following ordinary CS+ (red circle) and ordinary CS- (blue square) showed significant interaction of time and CS (F_(29,1305)_ = 9.88 p<0.001). Comparison at each time point revealed differences (p<0.01 with t-test) from 0.7 to 1.0s after juice onset (Fig 5E). Reverse trials during FSCV recordings were tried in four sessions (once in S, three times in C) and identical intense responses were observed after ordinary CS- in all of these sessions.

### 3–5. Responses to the prediction cue

The response to the prediction cue was detected in 11/19 recordings (Table 2). The difference between positive (red) and negative (blue) CS was detected only in 2/19 recordings (Table 2).

### 3–6. Local differences in juice reward responses

All of the successful results of liquid-reward trials are summarized in Table 2, and the results of successful simultaneous recordings at ventral striatum and putamen (5 from monkey C) were pooled (Fig 6). The interaction of time and recording location was not detected. The amplitude of responses in the putamen may be larger than ventral striatum, but not statistically significant (p = 0.06 for CS+). The responses are generally similar between ventral striatum and putamen. However, after CS- some recordings in the putamen showed positive responses while signal in the ventral striatum consistently decreased.

### 3–7. Response to food delivery

**Fig 7:**
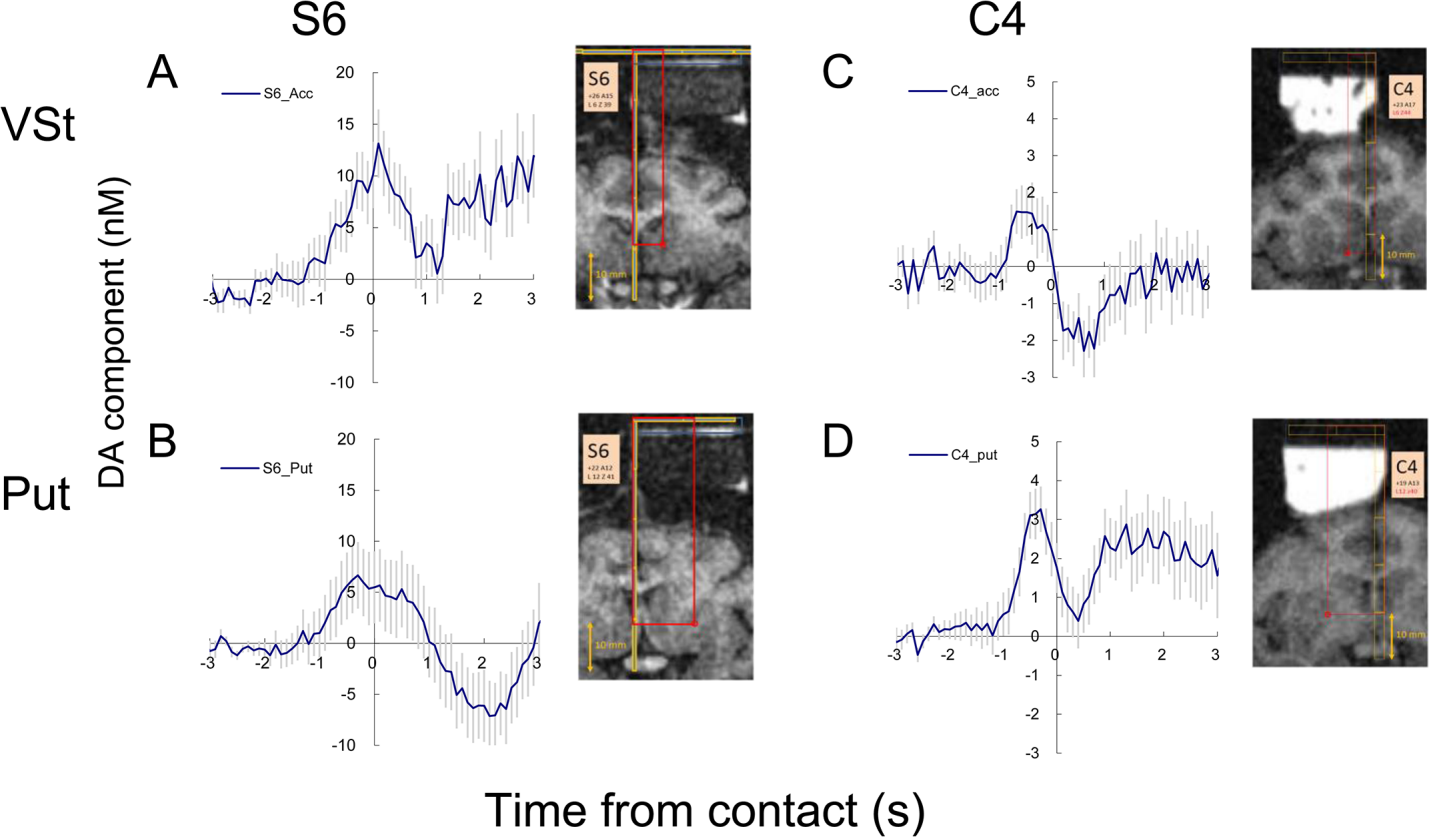
Examples of handed food reward responses in two monkeys (S and C). Simultaneous recording in ventral striatum (top) and putamen (bottom). The recorded positions are indicated on the MRI images. A small soft biscuit (Tamago-boro) was manually delivered by the examiner. The motion of the examiner turning back and picking up a biscuit from a table and putting it to the mouth of the monkey, occurred within 2 s. The data were aligned to the moment the biscuit touched the mouth. Note there is no physical/ingestional event before time 0, but only social observation. Vertical lines indicate s.e.m. S6 n = 24–25, C4 n = 46

A clear increase in the dopamine-like component was observed just before receiving the biscuit and a sharp drop after the retrievals (Fig 7). This result seems to reflect dopamine release associated with the expectation of food delivery. This reward-prediction response prior to the delivery was significant in 5/11 successful recordings (Table 2). In experiment S7, only a sharp drop after the acquisition of the food was observed (Table 2).

## Discussion

### 4–1. Detection of reward-induced dopamine release in the primate striatum

Dopamine release induced by electrical stimulation and reward signals was detected in the monkey striatum by FSCV on carbon fibers. Our technique is still preliminary and has occasional recording failures, but it detected event-related responses in more than half of the electrodes. Event-related temporal changes were detected on 19 carbon fibers in the striata of behaving monkeys. The simultaneous recordings in the ventral striatum and the putamen were successful in 8 experimental sessions in 2 monkeys. Positive responses to unexpected free reward and changes after a sudden reversal of positive and negative cues were evident, which indicate responses consistent with reward-prediction error. Smaller positive dopamine-like responses were detected at the timing cue signal and after juice delivery following a prediction cue. The dopamine release seems to be uniform between the striatal areas comparing to the result from rodents **[4]**.

Potential difference between ventral striatum and putamen were observed at the juice timing after CS- (blue square). The reward omission consistently induced a negative response of dopamine-like component at the ventral striatum while it was occasionally positive in a part of the recordings in the putamen. This contrast might be consistent with the positive spike response to the aversive event of dorso-lateral nigral dopamine neurons **[17]**, since putamen receives more projections from them.

Differential responses to the positive and negative cues were not detected in most cases. There are two possible reasons for these ambiguous cue responses. First, the response can be changed by training **[25, 26 and 27]** and our training may not have been sufficient for mature conditioned responses. Clear CS-induced midbrain dopamine responses need relatively extended training sessions, whereas the responses to a fixation cue can be observed early in training. The consistent response to the timing cue and poor CS responses in our results seem to be consistent with this observation of the early-to-middle stage of training **[26** **]**. On the other hand, the clear effect of CS reversal indicates some degree of discrimination learning between CS+ and CS-.

An evident effect of reward omission might be expected in the putamen after CS+ during the reversal session **[17]**, but the number of reversed sessions in our present study was too small for further statistical analysis. However, a sharp drop in the dopamine-like component was observed after the juice and food delivery. Recently, we have shown that the optogenetic suppression of dopamine release can cause a quick drop in the dopamine signal in mice **[20]**. The drop appears to be a reasonable response to a brief pause of dopaminergic activity.

### 4–2. Progress from the previous studies

For large experimental animals, there have been at least four voltammetric studies using carbon fiber. The pioneering work by Gerhardt **[28]** succeeded in detecting dopamine release by RPV (chronoamperometry) in anesthetized macaques by local KCl injection in Parkinsonian monkeys. Shon **[29]** and Gale **[30]** detected electrically evoked dopamine release in anesthetized pig and monkey brains, respectively. Ariansen (2012) **[2]** attempted to detect reward responses in the striatum of awake macaque monkeys using FSCV on carbon fibers but reward-induced dopamine responses were found only occasionally and marked pH responses were observed instead. Schulter (2014) **[3]** carefully studied the stimulation positions for evoked dopamine release in macaque monkeys and showed an example of a trial with reward response but did not perform statistical analyses of the behavioral effect.

Three technical reasons can be noted for our success in the detection of reward responses in the monkey brains: 1. statistical analysis, 2. animal dependency, 3. electrode sensitivity. First, since the electrochemical signals were small, statistical averaging is essential. We found that analysis of each individual noisy data was not very reliable, either by examination of the color plot matrix or PCR analysis. Therefore, we first applied PCR to individual recordings from the same trial type and averaged the dopamine-like components for further comparison between trial types (i.e. CS+ vs CS- cued trials). A similar result would also be available through averaging background-subtracted current first (Figs 4A, 5A and 5C) and then applying PCR to the averaged color plot. The latter procedure is preferable for reliable PCR analysis, but it makes an n = 1 which is not suitable for conventional ANOVA.

Second, in primate behavioral studies it is not unusual that the result can depend highly on the individual animal and their behavioral training. This may have contributed to the patterns of dopamine signal during juice reward task of monkey S that differed from expected outcomes. Therefore, we took steps in our behavioral procedures to further ensure the dopaminergic nature of the detected signal. First, free reward (no CS), regularly used in finding reward-responding nigral neurons in unit recording studies, and was effective to induce the response in dopamine-like component. Similarly, reversal of CS+ and CS- was also effective in enhancing the reward response.

It is repeatedly reported that dopamine responses are enhanced due to novel stimuli, in both unit recording **[25, 26]** and voltammetry **[27]**. This was the reason we did not train 50% reward trials in advance, but the sudden introduction of 50% trials probably confused the conditioned behavior of monkey S (Table 1). In our study, it was hard to detect liquid reward responses in monkey S, but the animal responded to the food reward clearly. If a similar animal was used and only liquid reward task was applied, it would greatly lower the confidence in the detection of the dopamine responses. Therefore, we recommend introducing qualitatively different rewards in cases where reliable signals are scarce in the original task.

Finally, we used relatively long carbon fiber tips for high sensitivity, although the fibers are prone to snap as this length. In spite of initial high sensitivity, the sensitivity of some carbon fibers deteriorated in the monkey brain. In our early detection of MFB responses, accumulation of tissue debris at the blunt tip of the silica tube (Fig 1A left) was noticed. Carbon fibers covered with tissue debris showed low dopamine sensitivity while pH sensitivity was unchanged. The carbon fibers supported by tapered glass capillary (Fig 1A right) seem to have less tissue adhesion, although the deterioration was not perfectly avoided. Compared to our experience in mice **[19, 20, 22]**, sensitivity loss occurred more often in the monkey brain. We suspect that tissue debris at the tip of the guide cannula is most likely the cause of tissue adhesion and sensitivity loss. The sensitivity loss occurred often in our early sessions in monkey S, but it became rare later, as we developed techniques for gentle handling of guide cannula and carbon fibers.

### 4–3. Further technical improvements for future studies

Although our study made some advancement, additional technical improvements will be needed for the next study. It will be important to determine whether the observed atypical results derived from true unknown type of dopamine responses or a technical failure (behavioral or electrochemical). Although we performed the customary analysis for validation of dopamine-derived signals, it remains possible that insufficient dopamine identification by PCR is the reason for these atypical responses. Validation of the dopamine template on each experiment by inserting a stimulation electrode for every experiment or implanting a chronic stimulation electrode would improve the reliability of the teaching template.

It is possible that the temporal delay of FSCV recording made it prone to overlook sharp responses similar to the known midbrain firing activity **[11,14,17,25 and 26]**. Voltammetric procedures with less temporal delay, such as RPV **[22]**, would help address this issue (S4 Fig). In vitro data have clearly shown the difference in temporal profile between voltammetric techniques **[1, 22 and 31]**. Our previous approach using constant-potential amperometry **[1]** was one good solution to avoid the deterioration of temporal delay in head-restricted preparations.

For recordings outside the striatum, the discrimination between monoamines should be verified. Enhancement of the evoked response by dopamine uptake inhibitor **[32]** (nomifensine, Fig 2) supports that the signal was mainly derived from catecholamine, but nomifensine is also known as norepinephrine uptake inhibitor. However, the monoamine tissue concentration in the rostral caudate of intact macaque monkey was reported that 7869 ng/g tissue for dopamine, 31 ng/g for norepinephrine, and 232 ng/g for serotonin, whereas the dopamine concentration of cerebral cortex was less than 100 ng/mg **[33]**. The extremely high concentration of dopamine in the monkey caudate suggests that dopamine is most likely to contribute to the major part of the response in this study.

Although the oxidation peak for serotonin in differential FSCV voltammograms (CV curve) is identical to dopamine, the reduction peak is different (S3 Fig), so the discrimination of serotonin and dopamine is possible by PCR analysis, only if both serotonin and dopamine template CV were available **[24]**. On the other hand, the discrimination of norepinephrine and dopamine seems to be more challenging because their CVs are identical. Unlike in vivo, there is no limitation in collection of various teaching template CV curves in vitro. Our estimation of dopamine changes using in-vivo and in-vitro teaching templates are roughly consistent (Fig 4), and changes in serotonin component showed similar but more sustained temporal changes to dopamine (S3 Fig). Additional templates of adenosine and serotonin influenced the PCR result and the estimated dopamine concentration was lower when four templates were applied instead of two (Figs 4E and S3A). Amplitude of serotonin was similar to dopamine in S3, but the serotonin result varied between recordings. The possibility of cross interference between dopamine and serotonin signals remains at present. Pharmacological and electrochemical confirmation is awaited for further segregation of serotonin and dopamine. In vitro template also allows estimation of electrochemically active molecules like adenosine **[34]** (S3 Fig). Our color plot (Fig 4A) and additional PCR analysis (S3C Fig) suggested changes in adenosine after CS+, but the reliability of such analysis has not been established yet. Constant-potential amperometry and RPV (S4 Fig) would be useful for the clear differentiation of dopamine signal from serotonin or adenosine **[1, 22]**.

Overall, dopamine release in response to behavioral reward events was successfully detected at multiple locations in the striatum of behaving monkeys. Voltammetric dopamine signals can reflect midbrain neuronal activity, local projection and presynaptic modifications. Further, the application of FSCV in the human brain has recently been attempted **[35]**. We believe that our result would be informative for such clinical trials. Continued development and variations of the approach we report here will be useful for advancement toward firmer conclusions on the behavioral correlates of the primate dopamine system.

## Supporting Information

S1 Checklist Arrive Checklist. (PDF)

S1 Fig Toys for environmental enrichment in the home cage. Biting wood, plumber’s materials and kitchen tools are placed in the home cage as toys.
(TIF)

S2 Fig Excluded data. Two examples of excluded trial data during the juice-reward sessions are presented. Adjacent trials, two previous and two following, are also shown for comparison.
(TIF)

S3 Fig PCR with multiple chemical templates. The same data shown in Fig 4 was further applied for PCR analysis with four in vitro templates, dopamine, pH, adenosine and serotonin (5-HT). The result of dopamine (A), pH (B), adenosine (C) and serotonin (D). The waveforms of in vitro templates are indicated on the right.
(TIF)

S4 Fig Examples of RPV recording. RPV recording at putamen (A and B) was switched to FSCV (C and D) during C4. Responses to the CS trials (A and C) and free juice (B and D). The absolute current was lower in RPV, but sharp responses to timing cue (A) and free juice delivery (B) were observed. N = 9 to 19. Vertical lines in B and D show s.e.m.
(TIF)

S1 Table Excluded data. As shown in S2 Fig, trials with large current jump were omitted from further analysis. Total number of trials within a session was 54 to 113 for juice reward task, 33 to 37 for reversed task, and 20 to 48 for food reward task. The trial was omitted if the current change during the time window (-5.4 to +2.5s from juice onset/ CS offset) exceeded 5nA.
(DOC)

## References

[1] Yoshimi K, Naya Y, Mitani N, Kato T, Inoue M, Natori S, et al Phasic reward responses in the monkey striatum as detected by voltammetry with diamond microelectrodes.Neurosci Res. 2011; 71: 49–62. 10.1016/j.neures.2011.05.013 21645558

[2] Ariansen JL, Heien ML, Hermans A, Phillips PE, Hernadi I, Bermudez MA, et al Monitoring extracellular pH, oxygen, and dopamine during reward delivery in the striatum of primates Front Behav Neurosci. 2012; 6: 36 10.3389/fnbeh.2012.00036 22783176 PMC3389715

[3] Schluter EW, Mitz AR, Cheer JF, Averbeck BB. Real-time dopamine measurement in awake monkeys.PLoS One. 2014; 9: e98692 10.1371/journal.pone.0098692 24921937 PMC4055617

[4] Brown HD, McCutcheon JE, Cone JJ, Ragozzino ME, Roitman MF. Primary food reward and reward-predictive stimuli evoke different patterns of phasic dopamine signaling throughout the striatum.Eur J Neurosci. 2011; 34: 1997–2006. 10.1111/j.1460-9568.2011.07914.x 22122410 PMC3237906

[5] Roeper J. Dissecting the diversity of midbrain dopamine neurons.Trends Neurosci. 2013; 36: 336–42. 10.1016/j.tins.2013.03.003 23582338

[6] Chuhma N, Mingote S, Moore H, Rayport S. Dopamine neurons control striatal cholinergic neurons via regionally heterogeneous dopamine and glutamate signaling.Neuron. 2014; 81: 901–912. 10.1016/j.neuron.2013.12.027 24559678 PMC3933825

[7] Cacciapaglia F, Saddoris MP, Wightman RM, Carelli RM. Differential dopamine release dynamics in the nucleus accumbens core and shell track distinct aspects of goal-directed behavior for sucrose.Neuropharmacology. 2012; 62: 2050–2056. 10.1016/j.neuropharm.2011.12.027 22261383 PMC3433749

[8] Badrinarayan A, Wescott SA, Vander Weele CM, Saunders BT, Couturier BE, Maren S, et al Aversive stimuli differentially modulate real-time dopamine transmission dynamics within the nucleus accumbens core and shell.J Neurosci. 2012; 32: 15779–15790. 10.1523/JNEUROSCI.3557-12.2012 23136417 PMC3752139

[9] Voorn P, Vanderschuren LJ, Groenewegen HJ, Robbins TW, Pennartz CM. Putting a spin on the dorsal-ventral divide of the striatum.Trends Neurosci. 2004; 27: 468–474. 15271494 10.1016/j.tins.2004.06.006

[10] Björklund A, Dunnett SB. Dopamine neuron systems in the brain: an update.Trends Neurosci. 2007; 30:194–202. 17408759 10.1016/j.tins.2007.03.006

[11] Schultz W. Behavioral dopamine signals.Trends Neurosci. 2007; 30: 203–210. 17400301 10.1016/j.tins.2007.03.007

[12] Graybiel AM. Habits, rituals, and the evaluative brain.Annu Rev Neurosci. 2008; 31: 359–387. 10.1146/annurev.neuro.29.051605.112851 18558860

[13] Rice ME, Patel JC, Cragg SJ. Dopamine release in the basal ganglia. Neuroscience. 2011; 198: 112–137. 10.1016/j.neuroscience.2011.08.066 21939738 PMC3357127

[14] Joshua M, Adler A, Mitelman R, Vaadia E, Bergman, H. Midbrain dopaminergic neurons and striatal cholinergic interneurons encodethe difference between reward and aversive events at different epochs ofprobabilistic classical conditioning trials. J. Neurosci. 2008; 28: 11673–11684. 10.1523/JNEUROSCI.3839-08.2008 18987203 PMC6671303

[15] Zhang T, Zhang L, Liang Y, Siapas AG, Zhou FM, Dani JA. Dopamine signaling differences in the nucleus accumbens and dorsal striatum exploited by nicotine.J. Neurosci. 2009; 29: 4035–4043. 10.1523/JNEUROSCI.0261-09.2009 19339599 PMC2743099

[16] Zhang L, Doyon WM, Clark JJ, Phillips PE, Dani JA. Controls of tonic and phasic dopamine transmission in the dorsal and ventral striatum. Mol Pharmacol. 2009; 76: 396–404. 10.1124/mol.109.056317 19460877 PMC2713129

[17] Matsumoto M, Hikosaka O. Two types of dopamine neuron distinctly convey positive and negative motivational signals.Nature 2009; 459: 837–841. 10.1038/nature08028 19448610 PMC2739096

[18] Cheer JF, Heien ML, Garris PA, Carelli RM, Wightman RM. Simultaneous dopamine and single-unit recordings reveal accumbens GABAergic responses: implications for intracranial self-stimulation.Proc Natl Acad Sci U S A. 2005; 102: 19150–19155. 16380429 10.1073/pnas.0509607102 PMC1323210

[19] Natori S.; Yoshimi K.; Takahashi T.; Kagohashi M.; Oyama G.; Shimo Y. et al Subsecond reward-related dopamine release in the mouse dorsal striatum.Neurosci Res. 2009; 63: 267–72. 19367786 10.1016/j.neures.2008.12.011

[20] Danjo T, Yoshimi K, Funabiki K, Yawata S, Nakanishi S. Aversive behavior induced by optogenetic inactivation of ventral tegmental area dopamine neurons is mediated by dopamine D2 receptors in the nucleus accumbens.Proc Natl Acad Sci U S A. 2014; 111: 6455–6460. 10.1073/pnas.1404323111 24737889 PMC4036004

[21] Saleem KS, Logothetis NK, A Combined MRI and Histology Atlas of the Rhesus Monkey Brain in Stereotaxic Coordinates, 2nd Edition, 2012; Academic Press 10.1016/j.mri.2010.03.023

[22] Yoshimi K, Weitemier A. Temporal Differentiation of pH-Dependent Capacitive Current from Dopamine.Anal Chem. 2014; 86: 8576–8584. 10.1021/ac500706m 25105214

[23] Yoshimi K, Ren YR, Seki T, Yamada M, Ooizumi H, Onodera M, et al Possibility for neurogenesis in substantia nigra of parkinsonian brain.Ann Neurol. 2005; 58: 31–40. 15912513 10.1002/ana.20506

[24] Heien ML, Khan AS, Ariansen JL, Cheer JF, Phillips PE, Wassum KM, Wightman RM. Real-time measurement of dopamine fluctuations after cocaine in the brain of behaving rats. Proc. Natl. Acad. Sci. U S A. 2005; 102: 10023–10028. 16006505 10.1073/pnas.0504657102 PMC1177422

[25] Schultz W, Apicella P, Ljungberg T. Responses of monkey dopamine neurons to reward and conditioned stimuli during successive steps of learning a delayed response task.J Neurosci. 1993; 13: 900–13. 8441015 10.1523/JNEUROSCI.13-03-00900.1993 PMC6576600

[26] Takikawa Y, Kawagoe R, Hikosaka O. A possible role of midbrain dopamine neurons in short- and long-term adaptation of saccades to position-reward mapping.J Neurophysiol. 2004; 92: 2520–2529. 15163669 10.1152/jn.00238.2004

[27] Clark JJ, Collins AL, Sanford CA, Phillips PE. Dopamine encoding of Pavlovian incentive stimuli diminishes with extended training.J Neurosci. 2013; 33: 3526–3532. 10.1523/JNEUROSCI.5119-12.2013 23426680 PMC3595050

[28] Gerhardt GA, Cass WA, Hudson J, Henson M, Zhang Z, Ovadia A, et al In vivo electrochemical studies of dopamine overflow and clearance in the striatum of normal and MPTP-treated rhesus monkeys.J Neurochem. 1996; 66: 579–88. 8592127 10.1046/j.1471-4159.1996.66020579.x

[29] Shon YM, Lee KH, Goerss SJ, Kim IY, Kimble C, Van Gompel JJ, et al High frequency stimulation of the subthalamic nucleus evokes striatal dopamine release in a large animal model of human DBS neurosurgery.Neurosci Lett. 2010; 475: 136–140. 10.1016/j.neulet.2010.03.060 20347936 PMC2874873

[30] Gale JT, Lee KH, Amirnovin R, Roberts DW, Williams ZM, Blaha CD, et al Electrical stimulation-evoked dopamine release in the primate striatum.Stereotact Funct Neurosurg. 2013; 91: 355–63. 10.1159/000351523 24107983

[31] Venton B.J., Troyer K.P.; Wightman R.M. Response times of carbon fiber microelectrodes to dynamic changes in catecholamine concentration.Anal Chem. 2002; 74: 539–546. 11838672 10.1021/ac010819a

[32] Wightman RM, Amatore C, Engstrom RC, Hale PD, Kristensen EW, Kuhr WG, May LJ.Real-time characterization of dopamine overflow and uptake in the rat striatum.Neuroscience. 1988; 25: 513–523. 3399057 10.1016/0306-4522(88)90255-2

[33] Pifl C., Schingnitz G., Hornykiewicz O. Effect of 1-methyl-4-phenyl-1,2,3,6-tetrahydropyridine on the regional distribution of brain monoamines in therhesus monkey. Neuroscience 1991; 44: 591–605. 1754053 10.1016/0306-4522(91)90080-8

[34] Swamy B.E.; Venton B.J. Subsecond detection of physiological adenosine concentrations using fast-scan cyclic voltammetry.Anal. Chem. 2007; 79: 744–750. 17222045 10.1021/ac061820i

[35] Chang SY, Kimble CJ, Kim I, Paek SB, Kressin KR, Boesche JB, et al Development of the Mayo Investigational Neuromodulation Control System: toward a closed-loop electrochemical feedback system for deep brain stimulation.J Neurosurg. 2013; 119: 1556–1565. 10.3171/2013.8.JNS122142 24116724 PMC4001796

