# Correction: Reward-Induced Phasic Dopamine Release in the Monkey Ventral Striatum and Putamen

**DOI:** 10.1371/journal.pone.0135592

**Published:** 2015-08-26

**Authors:** Kenji Yoshimi, Shiori Kumada, Adam Weitemier, Takayuki Jo, Masato Inoue

Table 2 is incorrect. The authors have provided a corrected version of Table 2 here.

**Table 2:** Summary of event-related temporal changes of dopamine-like component. Summary of ANOVA results. Prediction cue responses (before juice delivery) were calculated for the time window of -2.9 to 0.0 s from juice onset, where the timing cue appeared from -2.5 to -1.5 s and discriminative CS appeared from -1.5 to 0.0 s. Juice responses were calculated for the time window of -0.4 to 2.5 s from juice onset. For food delivery analysis, -2.9 to 3.0 s from contact to the mouth. Blanks in the handed food column indicate not tested. The food response in the S5 putamen was discarded (x) because the unstable line connection of the putamen electrode at the end of the experiment. The effect of time before juice delivery indicates a temporal change in the response to the prediction cue, and the time x CS interaction (t x CS) indicates a differential response to CS+ (red) and CS- (blue). The effect of juice over time indicates a temporal change in the response to juice delivery, and the time x CS interaction (t x CS) indicates a differential response to the presence or absence of juice delivery.

		Before Juice (Prediction cue)		After juice (Juice after CS)		Free Juice	Handed Food	
		time	t x CS	time	t x CS			
Ventral Striatum	S5	***	*	-	-	-	***	
	S6	-	-	***	-	-	***	
	S7	-	-	-	**	-	###	
	S8	-	-	-	*	*	***	
	C1	-	-	-	-	**	-	
	C2	***	-	-	**	***		
	C4	***	-	*	-	***	*	
	C5	-	-	###	-	-	-	
	C6	***	*	-	***	***		
	C7	***	-	-	-	**		
Rate of positive response		5/10	2/10	2/10	4/10	6/10	4/7	
Putamen	S3	-	-	-	-	-		
	S5	*	-	-	-	*		X
	S6	-	-	-	*	-	*	
	S7	-	-	-	*	-	###	
	C2	***	-	-	-	**		
	C4	***	-	*	*	***	**	
	C5	**	-	-	-	***	##	
	C6	***	-	-	-	***		
	C7	***	-	-	***	***		
Rate of positive response		6/9	0/9	1/9	5/9	6/9	2/4	

*: p<0.05,

**: p<0.01,

***: p<0.001 for positive responses.

^#^: p<0.05,

^##^: p<0.01,

^###^: p<0.001 for negative responses.
