# Supplementary figures and images for: Reward-Induced Phasic Dopamine Release in the Monkey Ventral Striatum and Putamen
Source: PLoS One. 2015 Jun 25;10(6):e0130443. doi: 10.1371/journal.pone.0130443 (PMC4482386; doi:10.1371/journal.pone.0130443)

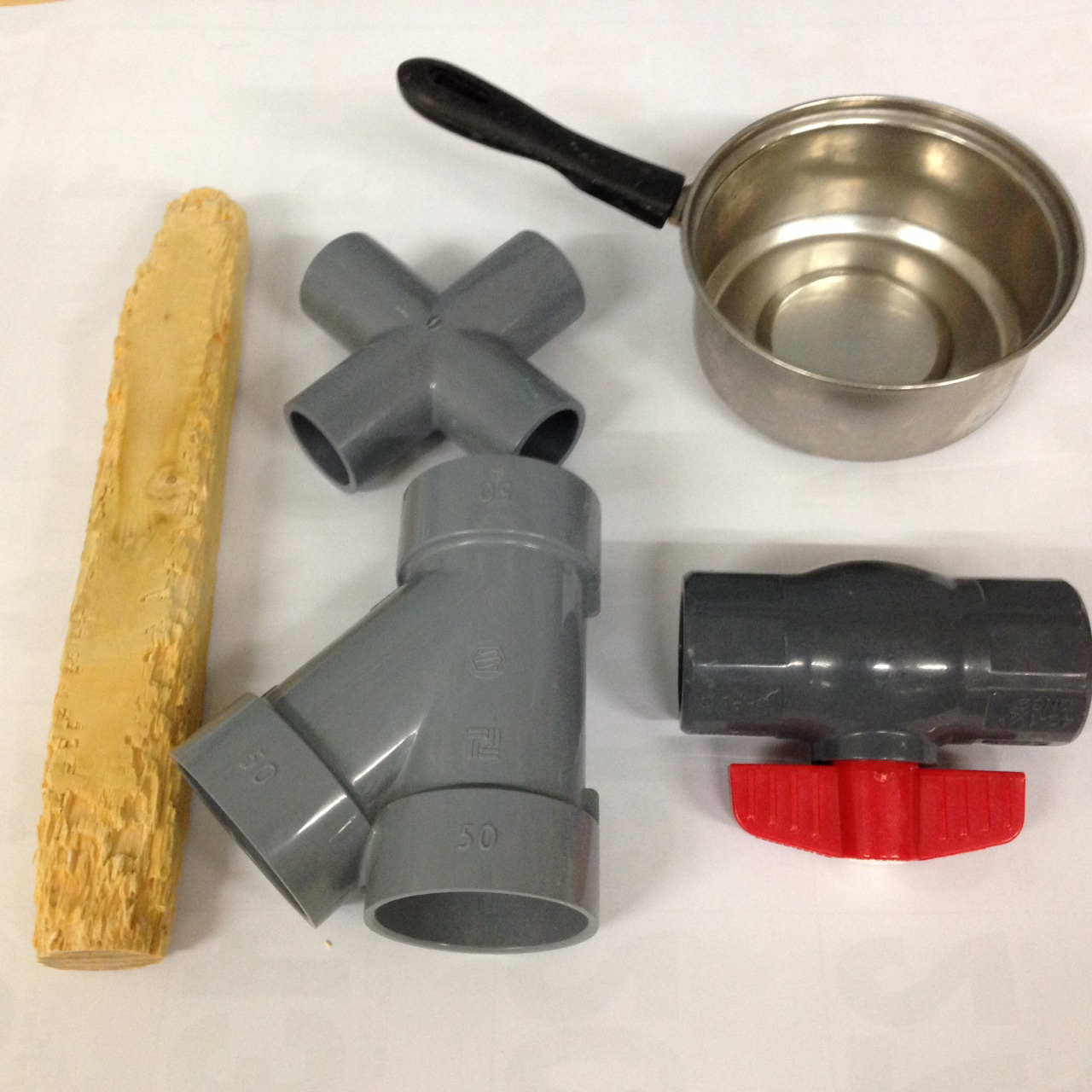

Supplement: S1 Fig — Biting wood, plumber’s materials and kitchen tools are placed in the home cage as toys. (TIF) [file pone.0130443.s002.tif]

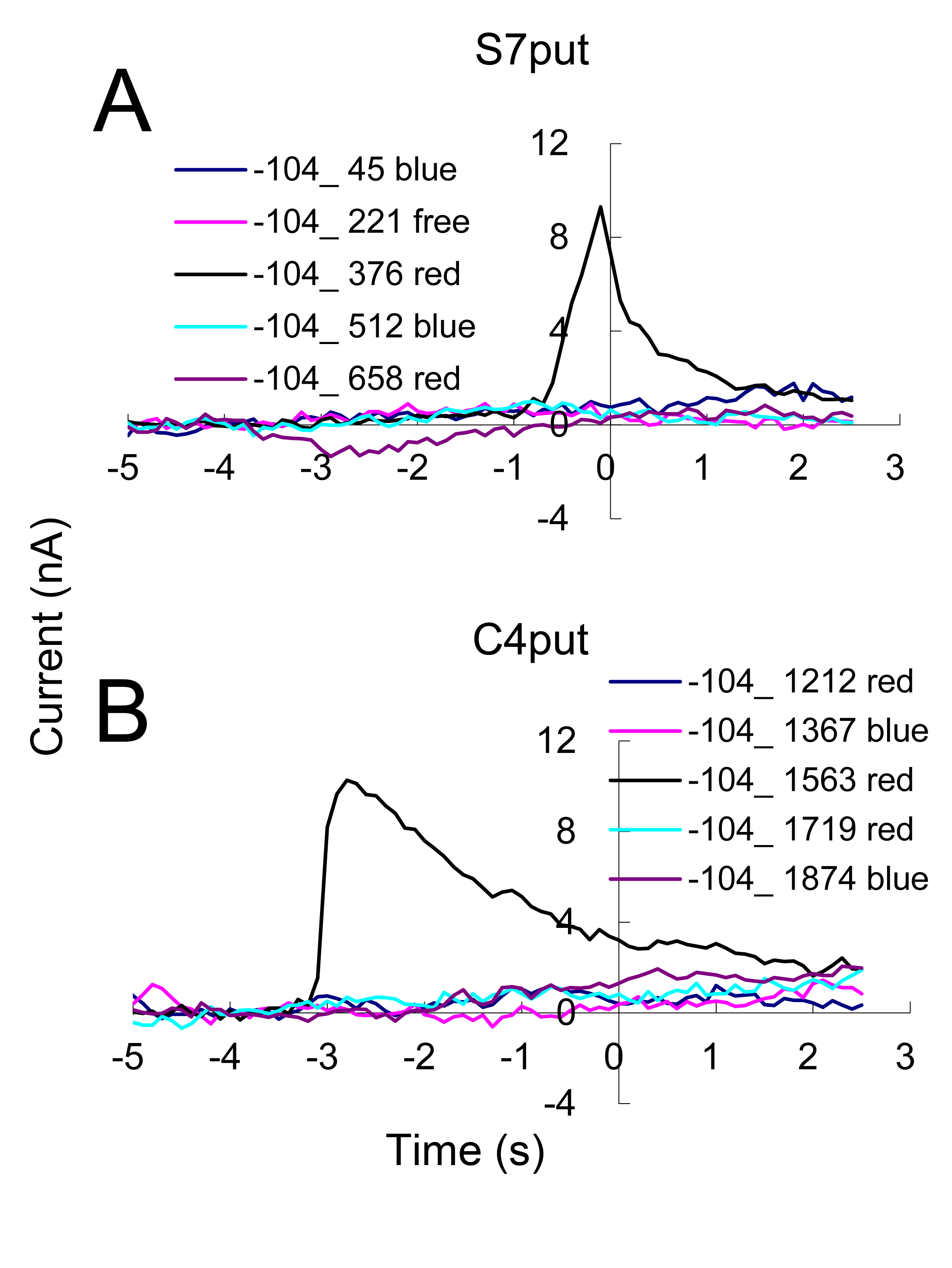

Supplement: S2 Fig — Two examples of excluded trial data during the juice-reward sessions are presented. Adjacent trials, two previous and two following, are also shown for comparison. (TIF) [file pone.0130443.s003.tif]

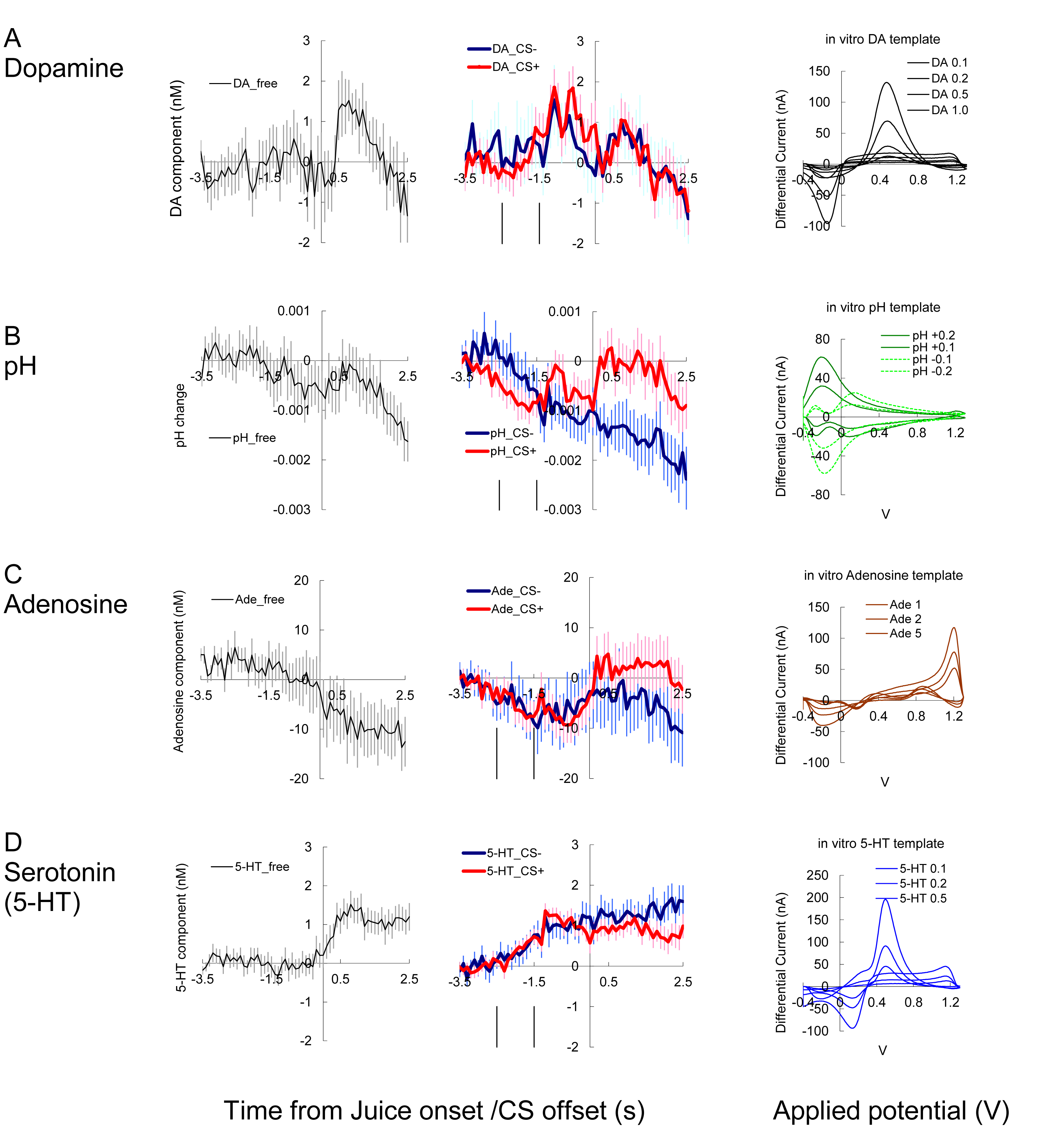

Supplement: S3 Fig — The same data shown in Fig 4 was further applied for PCR analysis with four in vitro templates, dopamine, pH, adenosine and serotonin (5-HT). The result of dopamine (A), pH (B), adenosine (C) and serotonin (D). The waveforms of in vitro templates are indicated on the right. (TIF) [file pone.0130443.s004.tif]

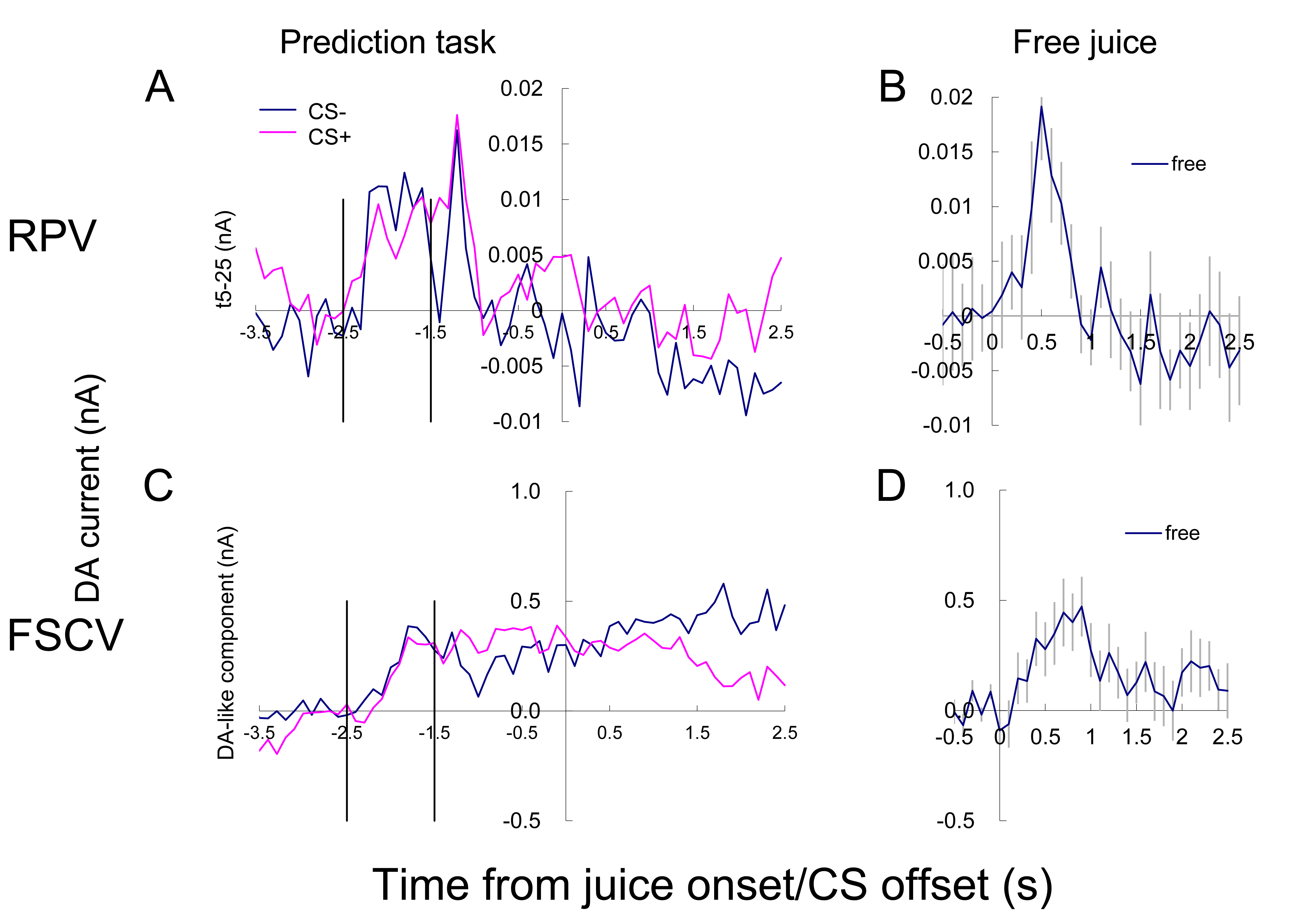

Supplement: S4 Fig — RPV recording at putamen (A and B) was switched to FSCV (C and D) during C4. Responses to the CS trials (A and C) and free juice (B and D). The absolute current was lower in RPV, but sharp responses to timing cue (A) and free juice delivery (B) were observed. N = 9 to 19. Vertical lines in B and D show s.e.m. (TIF) [file pone.0130443.s005.tif]
