# Supplementary material for: Reward-Induced Phasic Dopamine Release in the Monkey Ventral Striatum and Putamen
Source: PLoS One. 2015 Jun 25;10(6):e0130443. doi: 10.1371/journal.pone.0130443 (PMC4482386; doi:10.1371/journal.pone.0130443)
Supplement: S1 Table — As shown in S2 Fig, trials with large current jump were omitted from further analysis. Total number of trials within a session was 54 to 113 for juice reward task, 33 to 37 for reversed task, and 20 to 48 for food reward task. The trial was omitted if the current change during the time window (-5.4 to +2.5s from juice onset/ CS offset) exceeded 5nA. (DOC) [file pone.0130443.s006.doc]

Supporting Table ST1. Number of omitted trials (>5nA trials)
